# Intraspecific variation and phylogeographic patterns of the grey-capped greenfinch *Chloris sinica ssp*. (Passeriformes: Fringillidae)

**DOI:** 10.1080/23802359.2018.1491336

**Published:** 2018-08-08

**Authors:** Joo-Eun Kim, Jong-Gil Park, Kyoung-Soon Jin, Chungoo Park, Dong-Ha Nam

**Affiliations:** a Department of Biological Sciences, College of Natural Sciences, Chonnam National University, Gwangju, Korea;; b Birds Research Center, Korea National Park Research Institute, Korea National Park Service, Jeollanam-do, Korea;; c School of Biological Sciences and Technology, College of Natural Sciences, Chonnam National University, Gwangju, Korea

**Keywords:** Chloris sinica, Fringillidae, mitochondrial genome

## Abstract

To study the intraspecific variation of the grey-capped greenfinch *Chloris sinica* (Passeriformes: Fringillidae), we sequenced complete mitochondrial (mt) genome of the *C. sinica ssp.* prevalent in Ulleung Island, Republic of Korea. The full length of the genome is 16,812 bp, containing 37 genes (2 rRNAs, 13 proteins, and 22 tRNAs) with a putative control region (D-loop). A total of 98 single nucleotide polymorphisms (SNPs) in the full mt genome were retained for Ulleung Island population and these SNPs were greater than those of inland population compared to the reference China subspecies. The analysis of the SNPs revealed 18 SNPs for ND4 gene, with a dominant haplotype shared by Ulleung population compared to the reference China population. The phylogenetic analysis of the *C. sinica* subspecies showed that they are monophyletic, however, there is clear phylogenetic separation between China and Korea subspecies with strong support (100% bootstrap). These data will provide new insights into reconstruct the intraspecific phylogeographical patterns of *C. sinica* species.

The grey-capped greenfinch *Chloris sinica* (Passeriformes: Fringillidae) is a medium-sized (∼13–14 cm in length) finch that has been classified morphologically into five to six subspecies in East Asia: *ussuriensis* (geographical range: Northeastern China, Korea, and Eastern Siberia), *kawarahiba* (Kamchatka Peninsula, Kuril Islands, and Northeastern Hokkaido), *minor* (Japan: Southern Hokkaido to Kyushu, Honshu, and Shikoku; Korea: Jeju Island), *kittlitzi* (Bonin Islands including Iwo Jima), *sinica* (Central and Eastern China to Central Vietnam), and *chabarovi* (Mongolia to Northern Manchuria; del Hoyo et al. 2010). Based on the morphological characteristics and geographical distribution, it is assumed that three subspecies populations including *ussuriensis* (inland), *kawarahiba* (Ulleung Island), and *minor* (Jeju Island) are secluded geographically in Korean Peninsula (Won 1981; Won and Kim 2012). However, to date no phylogenetic analysis using molecular data for the *C. sinica* subspecies has been carried out.

For the phylogeographic analyses of *C. sinica*, we sequenced a new mt genome of *C. sinica ssp.* The sampled male *C. sinica ssp.* specimen was collected during the breeding season from Ulleung Island (120 km east of the Korean Peninsula). Total genomic DNA was extracted from the blood sample using the DNeasy Blood & Tissue kit (Qiagen, Valencia, CA) according to the manufacturer’s protocol. Twelve one PCR and sequencing primers specific to *C. sinica ssp.* were designed based on the previously reported mt genome sequences from *Carduelis sinica* (HQ915865 and KM078783). The remainder of blood sample was deposited at the Wildlife Specimen Bank in Chonnam National University, Korea.

The complete mt genome sequence of the specimen was 16,812 bp in length (GenBank accession No. MH047558), and contained 13 protein-coding, 22 transfer RNA (tRNA), and 2 ribosomal RNA (rRNA) genes, and a putative control (D-loop) region. All genes were encoded on the H-strand, except ND6 and eight tRNA genes (*tRNA^Gln^*, *tRNA^Ala^*, *tRNA^Asn^*, *tRNA^Cys^*, *tRNA^Tyr^*, *tRNA^Ser^*, *tRNA^Pro^*, and *tRNA^Glu^*) that were encoded on the L-strand. The base A + T of the mt genome is 55.5%, including 30.8% of A, 30.5% of C, 14.0% of G, and 24.7% of T. When compared to the available data (e.g. HQ915865), one and 98 sites were indels and singleton polymorphic sites (0.595% of the mitogenome sequences), respectively, including 74 and 5 were synonymous and non-synonymous SNPs, respectively. The remaining 19 SNPs were seen in noncoding regions. The total of 98 SNPs were retained for Ulleung Island population and these SNPs were greater than those of inland (36 SNPs from Taean; Kim et al. in press) subspecies compared to the reference China subspecies (HQ915865).

Furthermore, using entire mt genome sequences, we performed the phylogenetic analysis to determine the evolutionary relationships among subspecies of *C. sinica*. A neighbor-joining or maximum likelihood phylogenetic tree resolves three distinct clades with significant bootstrap support (100%): China (HQ915865), and inland Korea (KM078783 and MH047559), and island Korea (MH047558; this study; Figure 1). These clades clearly correspond to the three geographic regions sampled, which is inconsistent with previous morphological studies (Won 1981; Won and Kim 2012), wherein *C. sinica* populations are separated geographically between inlands (as a subspecies *ussuriensis* from China and Korea) and islands (*minor* from Jeju Island and *kawarahiba* from Ulleung Island).

**Figure 1.**
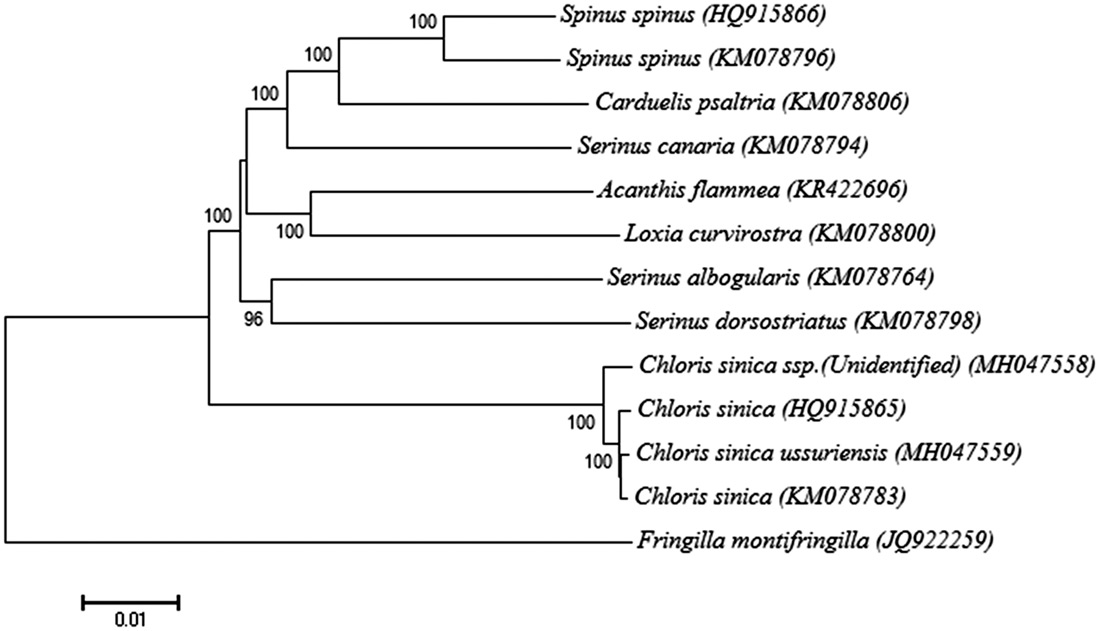
Phylogenetic tree of *Chloris sinica ssp.* and other related species belonging to Fringillidae based on complete mitochondrial genome sequences. The complete mitochondrial genomes were downloaded from GenBank and the phylogenetic tree is constructed by a neighbour-joining method with 1000 bootstrap replicates containing the full genomes derived from Fringillidae. Fringilla montifringilla was used as an outgroup for tree rooting. The percentage of replicate trees in which the associated taxa clustered together in the bootstrap test (1000 replicates) are shown next to the branches. The tree is drawn to scale, with branch lengths in the same units as those of the evolutionary distances used to infer the phylogenetic tree. GenBank accession numbers of each mitochondrial genome sequence are given in the bracket after the species name (MH047558 in this study collected from Ulleung Island, Korea; HQ915865 from China; MH047559 from Taean, Korea; KM078783 from unknown sampling site, Korea). The subspecies from Ulleung Island population in South Korea was approximated morphologically as *kawarahiba* (Won 1981; Won and Kim 2012), however to date no phylogenetic data for the *C. sinica* subspecies are available [hereinafter referred to as unidentified subspecies for Ulleung population(s)].

The *C. sinica* has been subdivided morphologically into three subspecies in Korean Peninsula: *ussuriensis* (inland), *kawarahiba* (Ulleung Island), and *minor* (Jeju Island). The phylogenetic relationship indicated that both inland and island populations are monophyletic, but there are two distinct geographical genetic structures between inland and island subspecies in Korea. Further research is required to differentiate the morphological variations and genetic clines in the *C. sinica* populations from a larger number of colonies including Jeju Island.
